# Some of the Ill Effects of Bromide of Potassium

**Published:** 1872-03

**Authors:** 


### Some of the Ill Effects of Bromide of Potassium.

"Under this heading Mr. T. O. Wood, Medical Superintendent of Dunston Lodge Asylum, publishes a paper, with cases, in the *British Medical Journal* of October 14, 1871, in which *inter alia*, he says its most dangerous effect is when, after a course of comparatively small doses, which do not seem to be taking any great hold of the system generally, or upon the mental symptoms, to control which it is given, it suddenly, and without apparent cause or warning, displays its cumulative effects, and rapidly reduces the patient to a condition of great bodily prostration, and completely alters the character of the mental symptoms. The physical prostration is at once evident. There is great muscular debility; dimness of sight with dilated pupils; irregular gait, the patient reeling as though intoxicated; whilst nausea, vomiting, or purging, with abdominal pain of a low aching character, may also be present, the breath having a peculiar disagreeable odor. Its effects upon the mental symptoms are no less marked. The patient, who was excited, glorying in his imaginary powers of body and mind, becomes despondent, sullen, melancholy, and frequently lachrymose, often even despairing.

In a previous number of the same Journal are cases reported by Witlow Bovis, L.R.C.P., and Dr. R. W. Foss, bearing out these statements, except in regard to the suddenness of the symptoms"—*New Remedies.*

CAUSE OF INFECTIOUS DISEASES.—Dr. Balfour also showed, Med. Chir. Soc. Edinburgh, a piece of lead soil-pipe which had been inserted into an iron pipe just below the seat of the water-closet. The iron had been corroded, and a way of escape thus made for the poisonous sewer gases into the house. This specimen had been removed from a house in which two fatal cases of diphtheria had occurred last summer; and Dr. Balfour stated that in his experience some such source of gaseous leakage had been invariably found, when sought for, in every house where diphtheria, typhoid fever, and other allied disorders had prevailed.—*Edinburgh Medical Journal, December,* 1871.

